# Comparison of azvudine, molnupiravir, and nirmatrelvir/ritonavir in adult patients with mild-to-moderate COVID-19: a retrospective cohort study

**DOI:** 10.1038/s41598-024-53862-y

**Published:** 2024-02-09

**Authors:** Mei-Ping Chen, Di-Xuan Jiang, Jia-Xi Rang, Hai-Bo Zhuo, Zhi-Guo Zhou

**Affiliations:** 1 Department of Respiratory and Critical Care Medicine, The Affiliated Changsha Hospital of Xiangya School of Medicine, Central South University (The First Hospital of Changsha), Changsha, 410000 People’s Republic of China; 2 Department of Infectious Disease, The Affiliated Changsha Hospital of Xiangya School of Medicine, Central South University (The First Hospital of Changsha), Changsha, 410000 People’s Republic of China; 3 Department of Nurse, The Affiliated Changsha Hospital of Xiangya School of Medicine, Central South University (The First Hospital of Changsha), Changsha, 410000 People’s Republic of China

**Keywords:** COVID-19, Azvudine, Nirmatrelvir/ritonavir, Molnupiravir, Retrospective study, Diseases, Infectious diseases, Drug therapy

## Abstract

This study aimed to explore the effectiveness and safety of azvudine, nirmatrelvir/ritonavir, and molnupiravir in adult patients with mild-to-moderate COVID-19. This retrospective cohort study included patients with mild-to-moderate COVID-19 (asymptomatic, mild, and common types) at the First Hospital of Changsha (Hunan Province, China) between March and November 2022. Eligible patients were classified into the azvudine, nirmatrelvir/ritonavir, or molnupiravir groups according to the antiviral agents they received. The outcomes were the times to nucleic acid negative conversion (NANC). This study included 157 patients treated with azvudine (n = 66), molnupiravir (n = 66), or nirmatrelvir/ritonavir (n = 25). There were no statistically significant differences in the time from diagnosis to NANC among the azvudine, molnupiravir, and nirmatrelvir/ritonavir groups [median, 9 (95% CI 9–11) vs. 11 (95% CI 10–12) vs. 9 (95% CI 8–12) days, *P* = 0.15], time from administration to NANC [median, 9 (95% CI 8–10) vs. 10 (95% CI 9.48–11) vs. 8.708 (95% CI 7.51–11) days, *P* = 0.50], or hospital stay [median, 11 (95% CI 11–13) vs. 13 (95% CI 12–14) vs. 12 (95% CI 10–14) days, *P* = 0.14], even after adjustment for sex, age, COVID-19 type, comorbidities, Ct level, time from diagnosis to antiviral treatment, and number of symptoms. The cumulative NANC rates in the azvudine, molnupiravir, and nirmatrelvir/ritonavir groups were 15.2%/12.3%/16.0% at day 5 (*P* = 0.858), 34.8%/21.5%/32.0% at day 7 (*P* = 0.226), 66.7%/52.3%/60.0% at 10 days (*P* = 0.246), and 86.4%/86.2%/80.0% at day 14 (*P* = 0.721). No serious adverse events were reported. Azvudine may be comparable to nirmatrelvir/ritonavir and molnupiravir in adult patients with mild-to-moderate COVID-19 regarding time to NANC, hospital stay, and AEs.

## Introduction

COVID-19 is a pandemic infectious respiratory disease caused by the novel coronavirus SARS-CoV-2 that has caused a serious public health, social, and economic crisis worldwide^1^. COVID-19 infection has been confirmed in more than 767 million people worldwide by June 4, 2023, including more than 6.9 million deaths^2^, imposing a huge burden on the global socioeconomic and medical systems^3,4^. As many countries relaxed their lockdown policies in the past months, including China, the newly reported volatile increase in COVID-19 cases in China by the Chinese Center for Disease Control and Prevention (China CDC)^5^ might be due to the antibody escape and immunoneutralization of the Omicron variant from the constant mutation and evolution of COVID-19^6,7^. The second wave of the pandemic caused by the SARS-CoV-2 Omicron variant tends to occur 5–7 months after the first wave^8^. The social and economic burden of mild and moderate COVID-19 infection cannot be ignored, though severe cases represent a small proportion of the second wave in most countries^9^.

Antiviral therapy is recommended by the World Health Organization for reducing mortality in hospitalized patients with COVID-19 infection^10^. Previous studies showed the efficacy and safety of nirmatrelvir/ritonavir (Paxlovid®)^11–13^ and molnupiravir^14,15^, although there is still some controversy^16^. However, nirmatrelvir/ritonavir and molnupiravir may not be widely available for developing countries due to the high costs. A new antiviral drug that would be safe, effective, and easily available is urgently needed.

Azvudine (FNC) is a nucleoside analog that can cure COVID-19 through its anti-SARS-CoV-2 activity concentrated in the thymus and strengthening patient immunity^17^. A pilot randomized controlled trial (RCT) with a small sample size (n = 20) in China showed that azvudine could shorten the treatment duration of patients with mild symptoms, and therefore saving a great deal of medical resources^18^. The same conclusion was drawn in a multi-center RCT in Brazil^19^. On the contrary, a retrospective Chinese study suggested that nirmatrelvir/ritonavir was superior to azvudine regarding the time to the first nucleic acid negative conversion (NANC) and antiviral activity^20^. Even so, the benefits of the three available anti-COVID-19 drugs for Chinese adult patients with mild or moderate symptoms remain uncertain.

Therefore, this study aimed to explore the effectiveness and safety of the azvudine, nirmatrelvir/ritonavir, and molnupiravir in adult patients with mild-to-moderate COVID-19. The results could help the management of patients with COVID-19.

## Methods

### Study design and patients

This retrospective cohort study included patients with confirmed COVID-19 at the First Hospital of Changsha (Hunan Province, China) between March and November 2022.

The inclusion criteria were (1) 18–60 years of age, (2) tested positive [cycle threshold (Ct) value ≤ 40] for SARS-CoV-2 nucleic acids by reverse transcriptase polymerase chain reaction (RT-PCR), (3) diagnosed as asymptomatic, mild, or common type of COVID-19 in accordance with the “Diagnosis and Treatment Protocol for Novel Coronavirus Pneumonia (Trial Version 9)”, and (4) received azvudine, molnupiravir, or nirmatrelvir/ritonavir as antiviral treatment. The exclusion criteria were (1) the time from diagnosis to treatment was > 5 days, (2) other antiviral therapies were given, or (3) incomplete key clinical information, such as the NANC time.

### Ethical statement

The study was approved by the ethics committee of the First Hospital of Changsha (Approval #KL-2020005). The requirement for individual informed consent was waived by the ethics committee of the First Hospital of Changsha because of the retrospective nature of the study. This study was conducted in accordance with the principles of the Declaration of Helsinki.

### Grouping and treatment

All eligible patients were grouped into the azvudine, nirmatrelvir/ritonavir, and molnupiravir groups according to their treatments. Azvudine was given at 5 mg/dose once daily for 7 days. Nirmatrelvir/ritonavir was given at 300 mg of nirmatrelvir and 100 mg of ritonavir twice a day for 5 days. Molnupiravir was given at 800 mg twice a day for 5 days. Other supportive treatments included ibuprofen and cough suppressant (Feilike Mixture).

### Data collection and outcomes

The baseline data collected included age, sex, COVID-19 type, comorbidities, number of COVID-19 symptoms, Ct value of the COVID-19 nucleic acid test, and the time from diagnosis to administration of the antiviral agents. The comorbidities included cardiovascular diseases, chronic lung diseases, diabetes, chronic liver and kidney diseases, tumors, acquired immunodeficiency syndrome (AIDS), and immune diseases with long-term glucocorticoids or immunosuppressive drugs. COVID-19 symptoms included fever, fatigue, cough, nasal congestion/runny nose, intolerance of cold/chills, shortness of breath, abdominal pain, diarrhea, headache, dizziness, palpitations, chest tightness, chest pain, dry/sore/itchy throat, muscle soreness, nausea/vomiting, dyspnea, gastric distension, stomach pain, loss of taste/poor appetite, and hyposmia.

The outcomes were the times to negative conversion in a COVID-19 nucleic acid test, including (1) the time from diagnosis to NANC, defined as the time from the first positive result in a nucleic acid test to the first NANC, (2) the time from the start of treatment to NANC, defined as the time from the first use of antiviral therapy to the first NANC, and (3) the NANC rates within 5, 7, 10 and 14 days after starting antiviral therapy. The length of hospital stay and adverse events (AEs) during hospitalization were recorded as well.

The Ct value of the SARS-CoV-2 nucleic acid test was detected through a nasopharyngeal swab. Per Chinese policy guidelines, patients underwent nucleic acid testing every other day until their Ct reached 30 or higher, after which testing became daily. A Ct value > 40 was considered negative. Laboratory test results were checked at admission and before discharge to monitor patients’ liver function. Discharge was granted only after two consecutive Ct readings exceeded 35, taken 24 h apart.

### Statistical analysis

R version 4.2.1 (The R Project for Statistical Computing, www.r-project.org) was used for statistical analysis. Age was expressed as mean ± standard deviation. The Ct value of the nucleic acid test was expressed as median (range). The comparison among the three groups was performed through one-way ANOVA or the Kruskal–Wallis H test. The categorical data were expressed as n (%), and the chi-square test was used to compare the three groups. The Kaplan–Meier method was used to analyze the time from diagnosis to the first NANC, the time from the start of antiviral therapy to NANC, and the length of hospital stay, calculating the median time and the corresponding 95% confidence interval (CI). The log-rank test was performed to analyze the differences among groups. Univariable and multivariable Cox regression analyses were performed to determine the association of the selection of azvudine, molnupiravir or nirmatrelvir/ritonavir with the NANC times and hospital stay. The multivariable analyses were adjusted for sex, age, COVID-19 type, comorbidities, Ct level, time from diagnosis to antiviral treatment, and number of symptoms. Variables with *P* < 0.05 in the univariable analyses were included in the multivariable analysis. Two-sided *P*-values < 0.05 were considered statistically significant.

## Results

### Characteristics of the patients

This study included 157 patients with COVID-19 treated with azvudine (n = 66), molnupiravir (n = 66), or nirmatrelvir/ritonavir (n = 25). The mean age of patients in the azvudine, molnupiravir, and nirmatrelvir/ritonavir groups were 41.1 ± 10.0, 38.0 ± 10.8, and 44.3 ± 13.4 years, respectively. A higher proportion of patients in the nirmatrelvir/ritonavir group had comorbidities (azvudine: 21.2%; molnupiravir: 48.5%; nirmatrelvir/ritonavir: 84.0%; *P* < 0.001). More asymptomatic patients were in the azvudine and molnupiravir groups (azvudine: 33.3%; molnupiravir: 37.9%; nirmatrelvir/ritonavir: 12.0%; *P* = 0.004). More patients started antiviral treatment on the day of diagnosis in the azvudine group than the molnupiravir or nirmatrelvir/ ritonavir groups (57.6% vs. 33.3% vs. 44.0%, *P* = 0.029). There were no statistically significant differences among groups regarding sex, Ct values, and number of symptoms (all *P* > 0.05) (Table 1).

**Table 1:** Characteristics of the patients.

	Azvudine (n = 66)	Molnupiravir (n = 66)	Nirmatrelvir/ ritonavir (n = 25)	*P*
Age, mean ± SD	41.1 ± 10.0	38.0 ± 10.8	44.3 ± 13.4	0.040
Sex (male), n (%)	46 (69.7%)	35 (53.0%)	18 (72.0%)	0.084
Comorbidities, n (%)	14 (21.2%)	32 (48.5%)	21 (84.0%)	< 0.001
COVID-19 type, n (%)				0.004
Asymptomatic	22 (33.3%)	25 (37.9%)	3 (12.0%)	
Mild	44 (66.7%)	32 (48.5%)	19 (76.0%)	
Common	0 (0%)	9 (13.6%)	3 (12.0%)	
Ct value, median (range)	21.7 [2.77, 37.6]	19.1 [1.25, 35.5]	22.4 [0.017, 40.0]	0.082
Level				0.668
(0,20]	28 (42.4%)	38 (57.6%)	10 (40.0%)	
(20,25]	15 (22.7%)	10 (15.2%)	6 (24.0%)	
(25,30]	11 (16.7%)	9 (13.6%)	4 (16.0%)	
[30, +	12 (18.2%)	9 (13.6%)	5 (20.0%)	
Number of symptoms				0.505
0	26 (39.4%)	24 (36.4%)	6 (24.0%)	
1	9 (13.6%)	9 (13.6%)	1 (4.0%)	
2	11 (16.7%)	11 (16.7%)	7 (28.0%)	
≥ 3	20 (30.3%)	22 (33.3%)	11 (44.0%)	
Time from diagnosis to antiviral treatment				0.029
0	38 (57.6%)	22 (33.3%)	11 (44.0%)	
1	27 (40.9%)	37 (56.1%)	13 (52.0%)	
≥ 2	1 (1.5%)	7 (10.6%)	1 (4.0%)	

### Clinical outcomes

**Figure 1:**
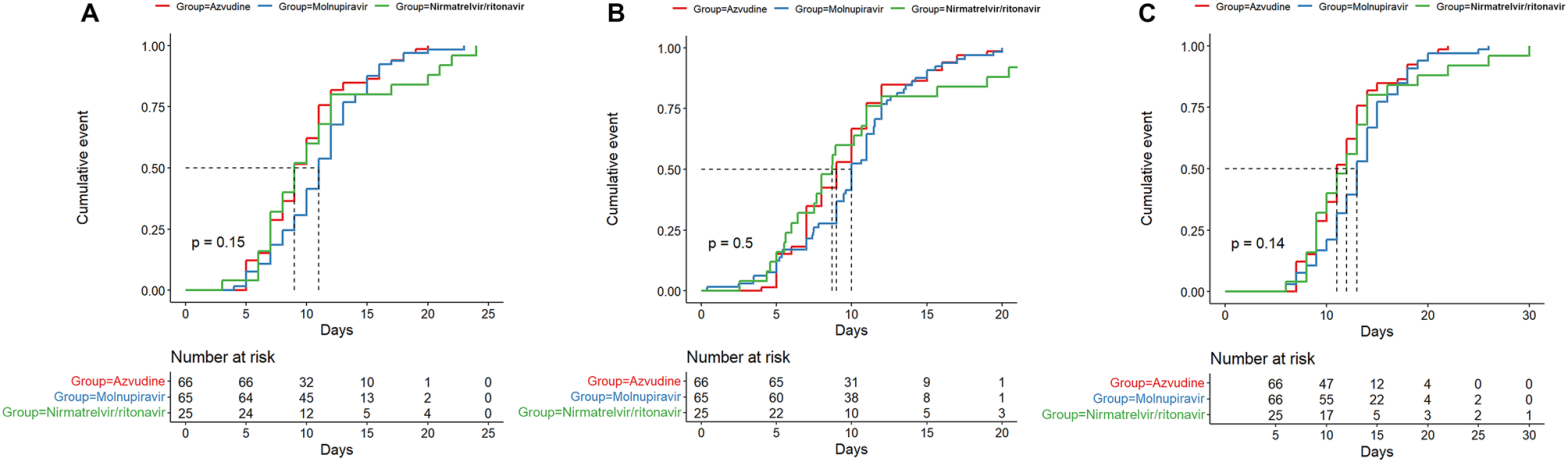
Kaplan–Meier curves of the clinical outcomes. (**A**) Time from diagnosis to nucleic acid negative conversion. (**B**) Time from administration to nucleic acid negative conversion. (**C**) Hospital stay.

There were no statistically significant differences in the time from diagnosis to NANC among the azvudine, molnupiravir, and nirmatrelvir/ritonavir groups [median, 9 (95% CI 9–11) vs. 11 (95% CI 10–12) vs. 9 (95% CI 8–12) days, *P* = 0.15] (Fig. 1A). No statistically significant differences were observed among the three groups in terms of the time from antiviral treatment to NANC [median, 9 (95% CI 8–10) vs. 10 (95% CI 9.48–11) vs. 8.708 (95% CI 7.51–11) days, *P* = 0.50] (Fig. 1B). The length of hospital stay were similar among the three groups as well [median, 11 (95% CI 11–13) vs. 13 (95% CI 12–14) vs. 12 (95% CI 10–14) days, *P* = 0.14] (Fig. 1C).

The cumulative NANC rates in the azvudine, molnupiravir, and nirmatrelvir/ritonavir groups were 15.2%, 12.3%, and 16.0% at day 5 (*P* = 0.858), 34.8%, 21.5%, and 32.0% at day 7 (*P* = 0.226), 66.7%, 52.3%, and 60.0% at 10 days (*P* = 0.246), and 86.4%, 86.2%, and 80.0% at day 14 (*P* = 0.721) (Table 2).

**Table 2:** Nucleic acid negative conversion rates from starting antiviral treatment.

Negative conversion rate, % (95% CI)	Azvudine (n = 66)	Molnupiravir (n = 66)	Nirmatrelvir/ritonavir (n = 25)	*P*
Within 5 days	15.2 (6.1–23.4)	12.3 (4.0–20.0)	16.0 (0.3–29.2)	0.858
Within 7 days	34.8 (22.3–45.4)	21.5 (10.9–30.9)	32.0 (11.0–48.0)	0.226
Within 10 days	66.7 (53.1–76.3)	52.3 (38.5–63.0)	60.0 (35.4–75.2)	0.246
Within 14 days	86.4 (75.0–92.6)	86.2 (74.6–92.5)	80.0 (56.2–90.9)	0.721

CI, Confidence interval.

### Univariable and multivariable analyses

The univariable and multivariable Cox regression analyses showed that the selection of azvudine, molnupiravir or nirmatrelvir/ritonavir was not associated with the time from diagnosis to NANC, the time from treatment start to NANC, or the length of hospital stay after adjustment for sex, age, COVID-19 type, comorbidities, Ct level, time from diagnosis to antiviral treatment, and number of symptoms (all *P* > 0.05) (Table 3).

**Table 3:** Univariable and multivariable Cox regression analyses.

	Univariable analysis			Multivariable analysis*		
	HR	95% CI	*P*	HR	95% CI	*P*
Time from diagnosis to nucleic acid negative conversion						
Azvudine	Ref			Ref		
Nirmatrelvir/ritonavir	0.680	0.418–1.106	0.120	0.607	0.357–1.055	0.077
Molnupiravir	0.744	0.527–1.051	0.094	0.814	0.535–1.238	0.335
Time from administration to nucleic acid negative conversion						
Azvudine	Ref			Ref		
Nirmatrelvir/ritonavir	0.723	0.441–1.187	0.200	0.637	0.366–1.109	0.111
Molnupiravir	0.842	0.597–1.186	0.325	0.867	0.571–1.316	0.501
Hospital stay						
Azvudine	Ref			Ref		
Nirmatrelvir/ritonavir	0.721	0.445–1.167	0.183	0.680	0.392–1.178	0.168
Molnupiravir	0.726	0.515–1.023	0.067	0.828	0.544–1.261	0.380

*Adjusted by sex, age, COVID-19 type, comorbidities, Ct level, time from diagnosis to antiviral treatment, and number of symptoms.

HR < 1 represented the drug as a risk factor for a longer time of negative conversion or hospital stays for patients.

HR, Hazard ratio; CI, Confidence interval.

### Subgroup analysis of the NANC rates

Patients who received azvudine had higher NANC rates at day 5, 7, 10, and 14 when they started the antiviral drug on the day of diagnosing COVID-19 compared to those who started it later. Similarly, patients who received molnupiravir had higher NANC rates at days 5, 7, and 10 when starting the drug on the day of diagnosis, but not at day 14. On the other hand, patients who received nirmatrelvir/ritonavir showed an opposite trend: those who started the drug at least one day after diagnosis had higher NANC rates at days 5, 7, and 10 compared to those who started it on the day of diagnosis, while the rates were similar at day 14 (Table 4).

**Table 4:** Subgroup analysis of the nucleic acid negative conversion rates from starting antiviral treatment.

Nucleic acid negative conversion rate, % (95% CI)	Medication started on the day of diagnosis	Medication started one day after diagnosis
Azvudine, n	32	28
Within 5 days	18.4 (5.1–29.9)	10.7 (0.0–21.5)
Within 7 days	36.8 (19.5–50.5)	32.1 (12.4–47.4)
Within 10 days	68.4 (49.6–80.2)	64.3 (41.3–78.3)
Within 14 days	92.1 (76.6–97.3)	78.6 (56.5–89.5)
Molnupiravir, n	22	44
Within 5 days	13.6 (0.0–26.9)	11.6 (1.5–20.7)
Within 7 days	31.8 (9.3–48.8)	16.3 (4.5–26.6)
Within 10 days	63.6 (36.8–79.1)	46.5 (29.3–59.5)
Within 14 days	81.8 (55.9–92.5)	88.4 (73.5–94.9)
Nirmatrelvir/ritonavir, n	11	14
Within 5 days	9.1 (0.0–24.6)	21.4 (0.0–40.2)
Within 7 days	27.3 (0.0–49.4)	35.7 (5.0–56.5)
Within 10 days	54.5 (13.2–76.2)	64.3 (27.9–82.3)
Within 14 days	81.8 (36.3–94.8)	78.6 (41.6–92.1)

CI, Confidence interval.

### Changes in blood biochemical parameters and adverse events

**Figure 2:**
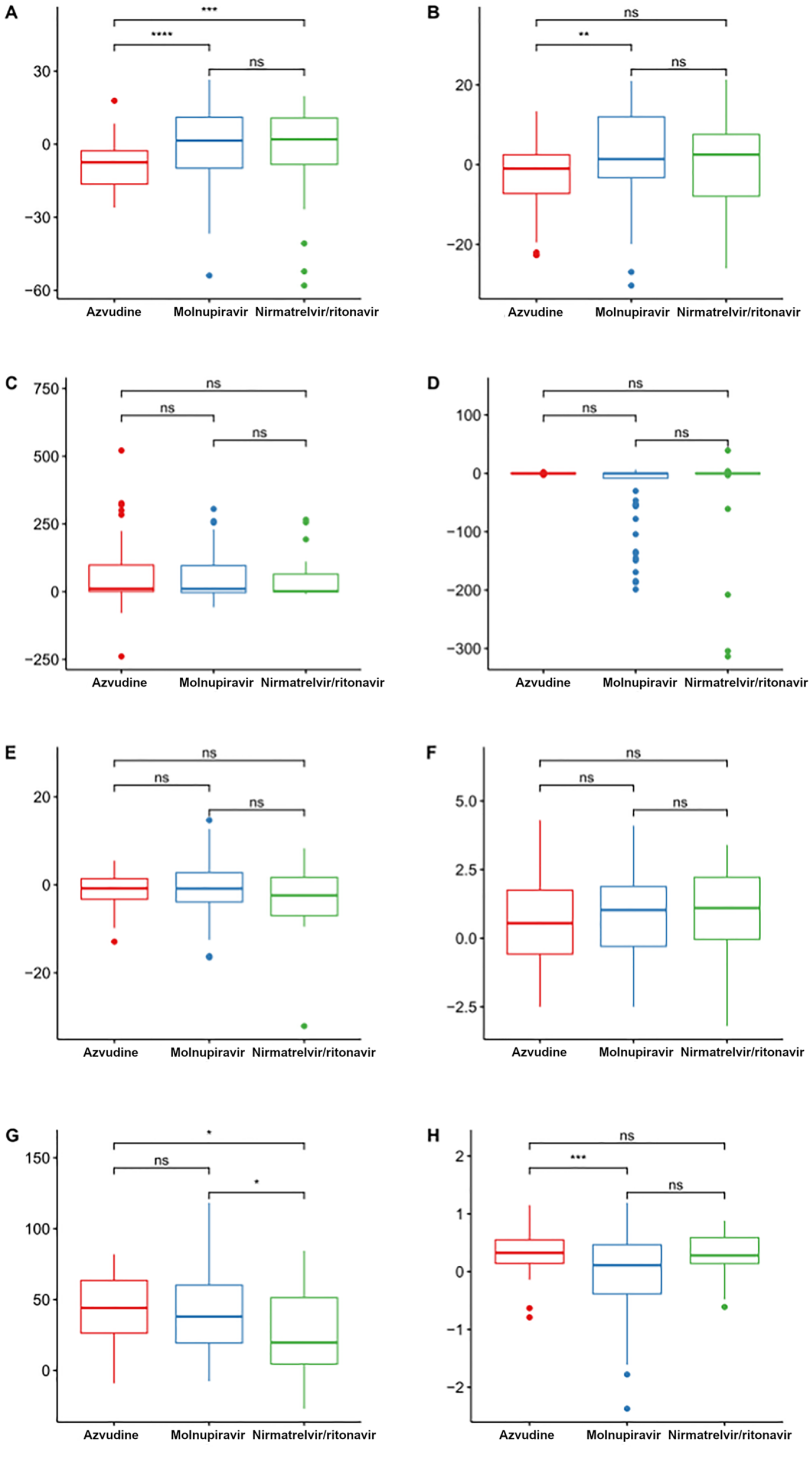
Changes in biochemistry indicators between admission and discharge. (**A**) Alanine aminotransferase. (**B**) Aspartate aminotransferase. (**C**) IgG. (**D**) IgM. (**E**) Globulin. (**F**) Blood urea nitrogen. (**G**) Creatinine. (**H**) Albumin to globulin ratio. ns indicates *P* > 0.05; * denotes *P* ≤ 0.05; ** indicates *P* ≤ 0.01; *** represents *P* ≤ 0.001; **** signifies *P* ≤ 0.0001.

The changes in biochemical parameters at discharge from admission were compared among the three groups (Fig. 2). The decreases in alanine aminotransferase (ALT) and aspartate aminotransferase (AST) levels were significantly greater with azvudine than with molnupiravir or nirmatrelvir/ritonavir (all *P* < 0.05), while there were no significant differences between molnupiravir and nirmatrelvir/ritonavir (both *P* > 0.05). The increase in creatinine in the nirmatrelvir/ritonavir group was significantly smaller than in the molnupiravir (*P* = 0.021) and azvudine (*P* = 0.017) groups; there were no significant differences between the molnupiravir and azvudine groups (*P* = 0.71). The increase in the albumin-to-globulin ratio of the azvudine group was significantly higher than in the molnupiravir group (*P* < 0.001), without difference with the nirmatrelvir/ritonavir group (*P* = 0.46); there were no significant differences between the nirmatrelvir/ritonavir and molnupiravir groups (*P* = 0.073). There were no significant differences among the three groups regarding the changes in IgG, IgM, globulin, and blood urea nitrogen (all *P* > 0.05).

No serious AEs (SAEs) were reported in any of the three groups. No AEs leading to suspension, dose reduction, or permanent discontinuation of the antiviral agents were observed.

## Discussion

This retrospective study explored the effectiveness and safety of azvudine, nirmatrelvir/ritonavir, and molnupiravir in adult patients with mild-to-moderate COVID-19. The results suggest no statistically significant differences among the three groups regarding the time from diagnosis to NANC, the time from starting antiviral treatment to NANC, or hospital stay. No SAEs were recorded in any group. Nevertheless, some numerical differences were observed among the three groups regarding the time to and the response patterns in time, suggesting that large-scale multicenter trials should be conducted to investigate these differences.

Previous studies indicated that nirmatrelvir/ritonavir can shorten the NANC time and hospital stay in patients with COVID-19^11,13,21^. It was also reported that molnupiravir could reduce the risks of hospitalization and death and shorten the time of viral clearance in adult patients with mild or moderate COVID-19^14–16,22^. Consequently, nirmatrelvir/ritonavir and molnupiravir are recommended in the World Health Organization guidelines for managing COVID-19^10^. Azvudine is an antiviral agent developed in China and has been shown to reduce the NANC time^18^. Regarding mild COVID-19, another study indicated that azvudine reduced the viral load and shortened the NANC time^19^. Still, most studies compared the antiviral drugs with a placebo or historical controls.

A recent retrospective study by Gao et al. revealed that nirmatrelvir/ritonavir in patients older than 65 years could better reduce the NANC time than azvudine^20^. In the present study, there were no differences between nirmatrelvir/ritonavir and azvudine in terms of NANC times, but the patients included here were mostly younger than in the previous study and had fewer comorbidities (and possibly fewer concomitant medications), as supported by a comparison between younger and older patients with COVID-19^23^. Furthermore, the groups were different in size, which could influence the comparisons. Some patients in Gao et al.^20^ were not prescribed antivirals at the initial phase, while in the present study, all patients started the antiviral drugs within 2 days of diagnosis, increasing the reliability of the present results. The present study suggests that azvudine in patients with mild-to-moderate COVID-19 was not weaker than nirmatrelvir/ritonavir. Still, the quality of evidence from retrospective studies is low, and confirmation is required.

Interestingly, the subgroup analysis suggested that the NANC rates with azvudine (at days 5, 7, 10, and 14) or molnupiravir (at days 5, 7, and 10) were higher if azvudine or molnupiravir were started on the same day as diagnosis, while an inverse pattern (at days 5, 7, and 10) was observed with nirmatrelvir/ritonavir, i.e., the NANC rate was higher if started 1–2 days after diagnosis. Still, the subgroups were too small to be able to perform reliable statistical analyses. Furthermore, such differences in NANC patterns were not reported before in the literature. Although there is a possibility that these results are coincidental, they could be explored in future large-scale multicenter studies.

In the present study, the Cox analyses showed that compared with azvudine, the HRs of molnupiravir or nirmatrelvir/ritonavir were all smaller than 1, suggesting a possible numerical benefit trend of azvudine, even after adjustment for sex, age, COVID-19 type, comorbidities, Ct level, time from diagnosis to antiviral treatment, and the number of symptoms. Still, all HRs were not statistically significant, and no conclusion can be reached at present, pending additional large-scale prospective studies.

Liver function impairment can be observed in patients with COVID-19 and could contribute to poor outcomes in patients with severe COVID-19 or those with pre-existing liver conditions^24–26^. Liver function impairment, particularly increased ALT and AST, caused by nirmatrelvir/ritonavir, has been reported^27–29^. On the other hand, changes in liver function impairment from molnupiravir have been reported to be relatively small^30^. In the present study, azvudine was the only drug to show significant decreases in ALT and AST levels from baseline. Still, the present study could not determine causality. It remains unknown whether azvudine does not increase ALT and AST and/or controls COVID-19 well enough to alleviate the liver injury. Still, potential concerns about liver function impairment may discourage some physicians and patients from using antiviral therapies, especially in mild or moderate COVID-19 associated with a small risk of poor outcomes. According to the present study, azvudine may be a safer option for patients with pre-existing liver function impairment, but studies must examine that point specifically. On the other hand, nirmatrelvir/ritonavir showed the smallest increases in creatinine levels compared with azvudine and molnupiravir. These results are a little surprising since azvudine has been suggested to be the drug of choice for patients with chronic kidney diseases compared with other antiviral drugs for COVID-19^31–33^. The discrepancy could be due to several factors, including patient selection and the characteristics of the patients. Additional studies are necessary to examine that issue.

No SAEs were recorded in the present study. Although a documentation bias is possible for retrospective studies, especially for mild AEs difficult to distinguish from the signs and symptoms of COVID-19 and in the context of stretched human healthcare resources, SAEs would have been noted since all included patients were hospitalized and continuously monitored for their signs and symptoms and aggravation. Furthermore, the antiviral therapies for mild or moderate COVID-19 are short (generally 5–7 days^10^), decreasing the risk of AE development. Favorable safety profiles have been noted for nirmatrelvir/ritonavir^11,13,21^, molnupiravir^14–16,22^, and azvudine^18,19^. Again, the small sample size and the retrospective nature of the study probably led to an underestimation of the AEs.

Recent studies suggested viral load rebounds during nirmatrelvir/ritonavir and molnupiravir treatment for COVID-19^34–37^, but no rebound phenomenon has been reported so far for azvudine. A dynamic analysis of the viral loads suggested that azvudine could effectively reduce the patient viral loads without rebound^19^. The exact causes of viral load rebound are unknown but are probably related to a resistance mutation arising through selection pressure. The differences in the targets and mechanisms of action between azvudine and the other drugs could lead to a lower risk of resistance mutation, but it will have to be examined in future studies.

This study has limitations. First, since this was a retrospective study, it may suffer from several biases, and some essential data might be missing, leading to patient exclusion. Second, this study involved a single center, leading to a small sample size, especially in the subgroups, preventing reliable analyses. Third, only patients with asymptomatic, mild or common type of COVID-19 were included, and more severe and complex cases were not. The interaction of antiviral drugs with other therapies used for severe COVID-19^38^ should be investigated. Furthermore, the non-random selection of drugs, qualitative nature of nucleic acid testing, and potential variance in patient viral loads can impact results interpretation. For more definitive outcomes, prospective randomized controlled trials comparing the efficacy of these drugs are necessary.

In conclusion, azvudine may be comparable to nirmatrelvir/ritonavir and molnupiravir in adult mild-to-moderate COVID-19 patients regarding NANC times, hospital stay, and AEs. Formal randomized controlled trials should be performed to compare the three drugs directly and to identify subgroups of patients who might benefit more from a specific drug.

## Data Availability

The datasets used and/or analysed during the current study are available from the corresponding author on reasonable request.
